# TTC30A and TTC30B Redundancy Protects IFT Complex B Integrity and Its Pivotal Role in Ciliogenesis

**DOI:** 10.3390/genes13071191

**Published:** 2022-07-01

**Authors:** Felix Hoffmann, Sylvia Bolz, Katrin Junger, Franziska Klose, Timm Schubert, Franziska Woerz, Karsten Boldt, Marius Ueffing, Tina Beyer

**Affiliations:** 1 Institute for Ophthalmic Research, Eberhard Karls University Tübingen, Elfriede-Aulhorn-Str. 7, 72076 Tübingen, Germany; f.hoffmann@uni-tuebingen.de (F.H.); sylvia.bolz@uni-tuebingen.de (S.B.); katrin.junger@uni-tuebingen.de (K.J.); franziska.klose@klinikum.uni-tuebingen.de (F.K.); timm.schubert@cin.uni-tuebingen.de (T.S.); franziska.woerz@uni-tuebingen.de (F.W.); karsten.boldt@uni-tuebingen.de (K.B.); marius.ueffing@uni-tuebingen.de (M.U.); 2 Werner Reichardt Centre for Integrative Neuroscience (CIN), University of Tübingen, Otfried-Müller-Str. 25, 72076 Tübingen, Germany

**Keywords:** cilia, IFT, TTC30 paralogues, CRISPR/Cas9, affinity proteomics, polyglutamylation

## Abstract

Intraflagellar transport (IFT) is a microtubule-based system that supports the assembly and maintenance of cilia. The dysfunction of IFT leads to ciliopathies of variable severity. Two of the IFT-B components are the paralogue proteins TTC30A and TTC30B. To investigate whether these proteins constitute redundant functions, CRISPR/Cas9 was used to generate single TTC30A or B and double-knockout hTERT-RPE1 cells. Ciliogenesis assays showed the redundancy of both proteins while the polyglutamylation of cilia was affected in single knockouts. The localization of other IFT components was not affected by the depletion of a single paralogue. A loss of both proteins led to a severe ciliogenesis defect, resulting in no cilia formation, which was rescued by TTC30A or B. The redundancy can be explained by the highly similar interaction patterns of the paralogues; both equally interact with the IFT-B machinery. Our study demonstrates that a loss of one TTC30 paralogue can mostly be compensated by the other, thus preventing severe ciliary defects. However, cells assemble shorter cilia, which are potentially limited in their function, especially because of impaired polyglutamylation. A complete loss of both proteins leads to a deficit in IFT complex B integrity followed by disrupted IFT and subsequently no cilia formation.

## 1. Introduction

Cilia and flagella are cellular organelles that are crucial in nearly all organisms and are involved in various developmental and physiological processes. Eukaryotic cilia can be grouped into primary non-motile and motile cilia. Consequently, cilia-associated functions such as signaling, ciliogenesis, gating and dynamic trafficking have been intensively studied. Ciliogenesis includes the assembly and disassembly of the cilia during the cell cycle. Due to the importance of these cellular functions, defects lead to more than 35 human diseases caused by the malfunction of cilia [1,2,3]. The assembly and maintenance of cilia are driven by the intraflagellar transport system (IFT). For cilium assembly, axonemal tubulins are imported from the cytoplasm and transported by IFT to the tip of the cilium where they are incorporated into the axoneme. IFT is a microtubule-based process which moves cargo along the axoneme [1]. The process of IFT involves the movement of large protein complexes with the help of motor proteins [4,5]. The anterograde transport is mediated by the motor protein kinesin-2 and retrograde movement by dynein. The IFT particles are organized in two subcomplexes called complexes A and B [6] consisting of 6 (A) and 16 (B) distinct proteins [7]. The architecture of IFT-B is well described and can be divided into peripheral and core subunits [8,9]. The stable IFT-B core complex is known as IFT-B1 (IFT88, -81, -74, -70, -52, -46, -27, -25, and -22) interacting with the second subcomplex known as IFT-B2 (IFT172, -80, -57, -54, -38, and -20) [10]. One of the core proteins is IFT70/TTC30, which is named for the two paralogues TTC30A and TTC30B. They contain a tetratricopeptide repeat (TPR) structural motif and eight of these motifs fold together to produce a single TPR domain. Such domains usually mediate protein–protein interactions and the assembly of multiprotein complexes [11,12]. TTC30A and B share highly similar nucleotide sequences (Supplementary Figure S1), which are conserved throughout several species [13]. Each paralogue is sufficient to support cilia assembly [14], and is able to interact with IFT-B complex components [8,15]. Further, Taschner et al. showed the direct interaction of the *Chlamydomonas reinhardtii* IFT70/DYF-1 orthologue with IFT52 [12,16]. DYF-1 depletion in *C. reinhardtii* leads to extremely shortened cilia, demonstrating its importance for IFT-B-mediated ciliary assembly [15]. This phenotype was also observed in zebrafish (*Danio rerio) *fleer** (*flr*, IFT70 orthologue) mutants, additionally linked to an ultrastructural defect in the axoneme. This defect likely results from a reduced level of glutamylated tubulin [17]. Polyglutamylated tubulin, a post-translational modification, was previously shown to be involved in stabilizing axonemes [18,19]. These findings suggest a role of *flr* in ciliogenesis as a structural component of IFT-B and a function as an IFT cargo adapter for a tubulin tyrosine ligase-like (TTLL) enzyme that catalyzes tubulin glutamylation [17,20]. The knockdown of cytoplasmic carboxypeptidase 5 (CCP5), restoring ciliary tubulin glutamylation in *flr* mutants and rescuing cilia assembly, indicates that polyglutamylation is a key driver of ciliogenesis [21].

In higher evolved organisms, such as mice or humans, TTC30 paralogues exist [8,22]. In the study presented here, it is our aim to investigate whether the human TTC30 paralogues TTC30A and TTC30B have redundant and/or individual functions in primary cilia. Single- as well as double-knockout cells were generated and primary cilia assembly, structural features and IFT-B complex stability were assessed. Additionally, interactome analysis using endogenously tagged cells for each paralogue separately was performed to define common or paralogue specific interactors, which would lead to a better understanding of the role of TTC30A or TTC30B. 

## 2. Materials and Methods

Antibiotics: ampicillin (Carl Roth, Karlsruhe, Germany), penicillin/streptomycin (Thermo Fisher Scientific, Waltham, MA, USA), and puromycin (Thermo Fisher Scientific, USA).

Antibodies: ARL13B (1:100; Proteintech, Munich, Germany), GAPDH (1:10,000; Cell Signaling, Danvers, MA, USA), GT335 (1:1500; Proteintech, Germany), IFT88 (1:200; Proteintech, Germany), IFT140 (1:200; Proteintech, Germany), Rootletin (1:250; SantaCruz, Heidelberg, Germany), TTC30 (1:100; SantaCruz, Germany), acetylated tubulin (1:2500; Sigma, Burlington, MA, USA), and γ-tubulin (1:500; Novus Biologicals, Littleton, CO, USA); secondary antibodies: Alexa Fluor 488/568 (1:350; Invitrogen, Waltham, MA, USA) and goat α rabbit/mouse antibodies (1:10,000; Jackson ImmunoResearch, Philadelphia, PA, USA).

Cell culture: Dulbecco’s Modified Eagle’s Medium (DMEM, Sigma-Aldrich, USA) and fetal bovine serum (Sigma-Aldrich, USA).

Immunoprecipitation: anti-Flag-M2-agarose beads (Sigma-Aldrich, USA) and Flag-peptide (Sigma-Aldrich, USA).

Immunofluorescence staining: PFA (Morphisto, Offenbach, Germany) and Fluoromount-G (Invitrogen, USA).

Cell lines: HEK293T (CRL-3216, ATCC) for immunoprecipitation and protein complex analysis; hTERT-RPE1 cells (CRL-4000, ATCC) efficiently assemble primary cilia suitable for localization studies and phenotype analysis.

### 2.1. Generation of Knockin and Knockout Cell Lines

**Figure 1:**
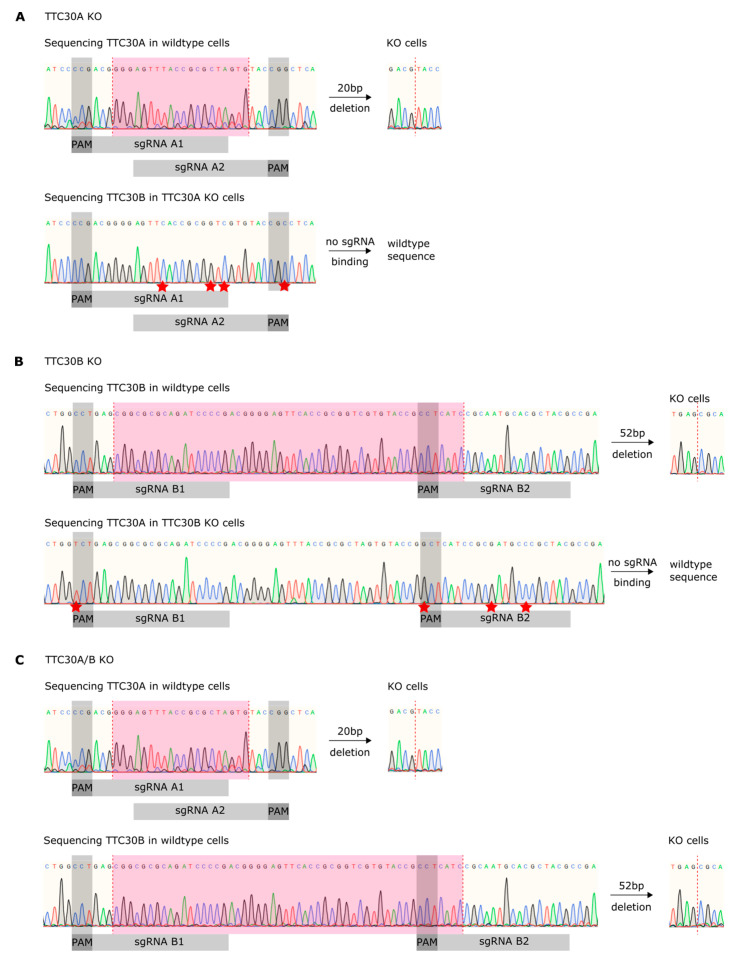
Shown are fragments of wildtype TTC30A/B genes along with PAM sequences (dark grey), sgRNA binding sites (light grey) and deleted base pairs (magenta) and sequencing results after CRISPR/Cas9-mediated KO generation. Sequence mismatches of sgRNAs are highlighted with red stars. (**A**) CRISPR/Cas9-mediated homozygous 20 bp deletion led to a premature stop codon. Specific TTC30A sgRNAs do not bind in the TTC30B gene, resulting in a specific KO. (**B**) CRISPR/Cas9-mediated homozygous 52 bp deletion led to a premature stop codon. Specific TTC30B sgRNAs are non-binding in TTC30A, resulting in a specific KO. (**C**) Mixing TTC30A and B specific sgRNAs resulted in homozygous TTC30A and B double KO.

The CRISPR/Cas9 system was used to generate specific TTC30A and/or TTC30B knockout cell lines (KO, hTERT-RPE1, HEK293T) as well as specifically introduce the FLAG-tag to the N-terminus of either TTC30A or TTC30B genes (HEK293T) via homology directed repair. sgRNAs were designed using CCTop software [23] and predicted targets were chosen according to their gene location (Figure 1 and  Figure S1). Additionally, off-targets were taken into consideration and the overall CRISPR/Cas9 efficiency score was determined [24]. Top and bottom strands were annealed and subsequently cloned into the Cas9 vector (pSpCas9(BB)-2A-Puro (PX459) V2.0; gift from Feng Zhang; Addgene; USA), followed by transformation into competent *Escherichia coli* DH5α bacterial cells via heat shock and plated onto ampicillin-supplemented (Carl Roth, Germany) LB-Agar dishes. Colonies were picked and transferred to selective LB-medium followed by DNA isolation according to the PureYield^®^ Plasmid Midiprep Protocol System (Promega, Madison, WI, USA). Cells were transiently transfected according to Lipofectamine™ 3000 reagent protocol (Thermo Fisher Scientific, USA). Selective puromycin (Thermo Fisher Scientific, USA) treatment followed by clonal selection was performed. Genomic DNA was extracted and purified. For verification of successful KO generation, the region of interest was amplified by PCR, the product was purified and sent for Sanger sequencing (Eurofins Scientific, Luxembourg). For rescue experiments, hTERT-RPE1 TTC30A/B double-KO cells were transfected with TTC30A or TTC30B constructs [8]. Neomycin resistance (NeoR) encoded by the rescue construct was used for antibiotic selection of stably transfected cells. Cells were treated for three weeks with 0.4 mg/mL geneticin disulfate-supplemented (G418, Carl Roth, Germany) DMEM. As control cells, hTERT-RPE1 (localization studies) and HEK293T (interactome identification) wildtype cells were treated accordingly but with an empty Cas9 vector containing no gene targeting sgRNA. All generated wildtype, KO and FLAG-tagged cell lines were treated with Dulbecco’s Modified Eagle’s Medium (DMEM, Sigma-Aldrich), supplemented with 10% fetal bovine serum (Sigma-Aldrich, USA) as well as 0.5% penicillin/streptomycin (Thermo Fisher Scientific) and incubated at 37 °C and 5% CO_2_.

### 2.2. Immunofluorescence Staining

Control and KO hTERT-RPE1 cells were used for ciliary length and intensity measurements as well as localization studies. At first a three-day serum-free treatment was necessary to induce cilia formation. Cells were then fixed using 4% PFA, permeabilized with 0.3% PBST and blocked by applying 10% normal goat serum in 0.1% PBST, followed by primary antibody and subsequent fluorescent secondary antibody incubation. Finally, cells were mounted with Fluoromount-G (Invitrogen) and examined via fluorescence microscopy.

Images were captured using a Zeiss Axio Imager Z1 ApoTome microscope (Carl Zeiss Microscopy GmbH, Munich, Germany). The setup includes an AxioCam MRm camera as well as 40× (NA 1.3) and 63× (NA 1.4) oil immersion objective lenses. Images were acquired as Z-stacks and processed using Zeiss ZEN 3.0 Blue Edition (Carl Zeiss Microscopy GmbH, Germany). The process of quantifying ciliary length was accomplished by using the NeuronJ plugin [25] for FIJI software [26]. Therefore, the ciliary channel of ApoTome RAW convert files was optimized for analysis by excluding background signal, identifying true cilia fluorescence signal, and increasing software recognition of cilia. Intensity measurements were also conducted with FIJI software. For this purpose, initial Z-stacks were acquired with identical exposure times, grey values and γ settings. Additionally, the tubulin intensity was also optimized by calculating the corrected total cell fluorescence. Statistical analysis (unpaired Student *t*-Test) was performed and graphs were created with GraphPad Prism version 5.0.1 for Windows, GraphPad Software (San Diego, CA, USA), www.graphpad.com, accessed on 22 June 2022.

### 2.3. SDS-PAGE and WesternBlot

For verification of KO cell lines and investigation of IFT proteins, SDS-PAGE using an 8% Tris-Glycine-based separation gel and running buffer was performed. Full wet tank blotting (Bio-Rad, Hercules, CA, USA) was followed by 5% BSA block as well as primary and secondary antibody incubation. Membranes were treated with ECLplus (Thermo Fisher Scientific, USA) and images were taken using the Fusion FX7 imaging system (Vilber, Collégien, France).

### 2.4. Affinity Purification

For interactome identification of TTC30A and TTC30B at the endogenous level, HEK293T control and FLAG-tagged TTC30A or TTC30B cells were serum starved for 16 h inducing ciliary assembly. For LCA5 and IFT88 interactome analysis by Strep affinity purification, Strep/FLAG tagged constructs were transfected into TTC30B, TTC30A/B KO and Cas9 control cells. Control Strep/FLAG-tagged RAF1 was used. After lysis, the protein concentration was measured using Bradford assay. Identical amounts of protein for each sample were incubated with anti-Flag-M2-agarose bead (Sigma-Aldrich, USA) suspension or Strep-Tactin Superflow (IBA, Göttingen, Germany) followed by several washing steps and elution of bound protein with Flag-peptide (Sigma-Aldrich, USA) or Strep elution buffer (IBA, Germany). A methanol–chloroform-based protein extraction was performed and subsequent trypsin digestion. The digested proteins were desalted via stop-and-go extraction tips (Thermo Fisher Scientific, USA) and prepared for mass spectrometry (MS) analysis.

### 2.5. Mass Spectrometry

For LC-MS/MS analysis, an Ultimate3000 nano-RSLC was coupled to a Fusion by a nanospray ion source. Prepared peptide mixtures were loaded onto a nanotrap column (µ-Precolumn 300 µm i.d. × 5 mm, packed with Acclaim PepMap100 C18, 5 µm, 100 Å; Dionex, Sunnyvale, CA, USA). Injection was conducted with a flow rate of 30 µL/min in 98% of buffer C (0.1% TFA in HPLC-grade water) and 2% of buffer B (80% ACN, 0.08% formic acid in HPLC-grade water). After 3 min, peptides were eluted and separated on an analytical column (75 µm × 25 cm, packed with Acclaim PepMap RSLC, 2 µm, 100 Å; Dionex, USA) at a flow rate of 300 nL/min with a linear gradient from 2% up to 30% of buffer B in buffer A (2% ACN, 0.1% formic acid) for 82 min after an initial step of 3 min at 2% buffer B. Remaining peptides were eluted with a steep gradient (30% to 95% in 5 min) followed by 5 min at constant 95% of buffer B before the gradient was decreased rapidly in 5 min to 2% of solvent B for the final 20 min. In the data-dependent analysis, full-scan MS spectra were measured on the Fusion in a mass-charge range from m/z 335–1500 with a resolution of 70,000. The ten most abundant precursor ions were selected with a quadrupole mass filter, if they exceeded an intensity threshold of 5.0 e4 and were at least doubly charged, for further fragmentation using higher-energy collisional dissociation (HCD) followed by mass analysis of the fragments in the iontrap. The selected ions were excluded for further fragmentation the following 20 s. Max Quant software 1.6.0.16 was used for label-free quantification (Supplementary Figure S2) with current SwissProt database and Perseus software 1.6.2.3 [27] for data and statistical analysis (Student *t*-Test; Significance A). Data are available from ProteomeXchange with identifier PXD034412.

## 3. Results

### 3.1. Single TTC30A or TTC30B Knockout Mildly Affects Ciliogenesis with No Effect on IFT Complex A or B Component Localization

To investigate a paralogue-specific function of TTC30A and B in ciliogenesis, knockout cells (KO) of retinal-pigmented epithelial cells (hTERT-RPE1) were generated using CRISPR/Cas9. The transfection of hTERT-RPE1 cells with TTC30A- or B-targeting sgRNAs was followed by single clone selection and DNA extraction. To specifically target TTC30A or B and to generate double-KO cells, deletions by combining two sgRNAs were induced. Using Sanger sequencing, deletions followed by an open-reading frameshift and predicted early stop codon were identified (Figure 1 and Figure S1). Furthermore, sequencing verified the successful generation of homozygous TTC30A and TTC30B single KO as well as a TTC30A/B double KO (Figure 1).

**Figure 2:**
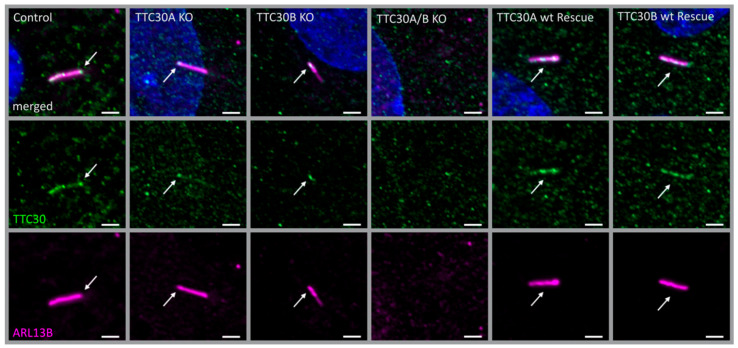
Fluorescent light microscopy pictures of hTERT-RPE1 control, TTC30A, TTC30B and TTC30A/B double-KO cells, TTC30A and TTC30B rescue (from left to right) are shown. The cells were stained for ARL13B (magenta) and TTC30 (green), DNA is marked in dark blue and co-localization shown in white. The scale bar measures 2 µm.

To analyze whether homozygous and specific hTERT-RPE1 TTC30A, TTC30B single- and TTC30A/B double-KO cells had an impact on ciliogenesis as well as on the localization of other IFT components, these cells were analyzed using immunofluorescence microscopy. TTC30 staining was performed to verify differences in protein abundance in wildtype versus mutant cell lines. Unfortunately, there is no antibody available to distinguish between TTC30A and TTC30B due to the amino acid sequence similarity. Still, a clear reduction in TTC30 signal intensity can be seen in single-KO compared to control cells (Figure 2). In the double-KO cells, no specific staining was detected. Similar results were observed for total protein level by Western blot (Supplementary Figure S4).

**Figure 3:**
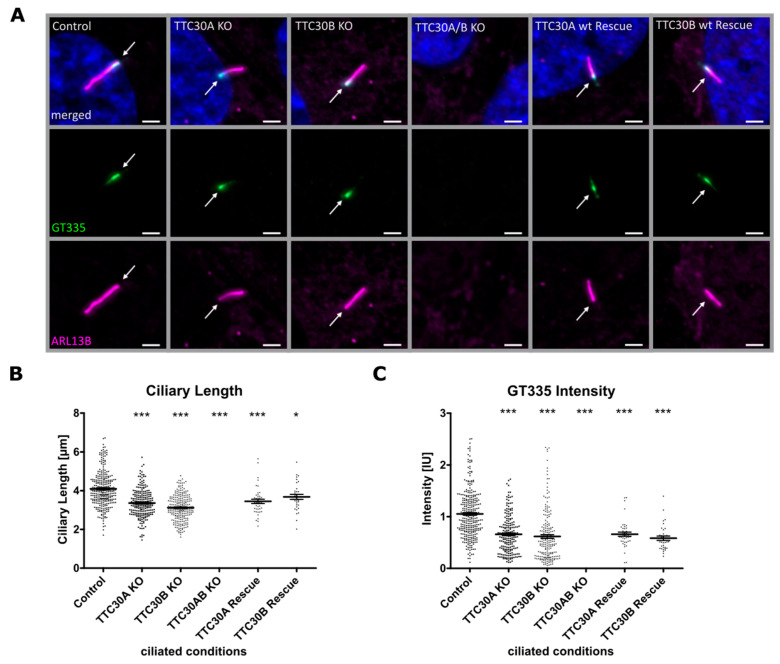
(**A**) Fluorescent light microscopy images of hTERT-RPE1 control, TTC30A, TTC30B, TTC30A/B double-KO cells, TTC30A and TTC30B rescue (from left to right) are shown. The cells were stained for ARL13B (magenta) and polyglutamylated tubulin (GT335, green), DNA is marked in dark blue and co-localization is shown as white. The scale bar measures 2 µm. In TTC30A/B, single- and double-KO as well as rescue cells’ ciliary length (**B**) was measured using ARL13B as an indicator for ciliary length. Measurement of fluorescent intensity of polyglutamylated tubulin staining is shown in (**C**). In the scatter dot plots, results gained in two independent experiments are shown with each dot representing one cilium. For statistical analysis, the mean was calculated. *p* values below 0.05 are represented by * and below 0.001 by ***. Error bars represent the s.e.m.

Next, cilia number and length were investigated using the ciliary protein ARL13B, which localizes to the ciliary membrane and can be used as a marker for these measurements [28] (Figure 2 and Figure 3). Furthermore, GT335 labeled polyglutamylated tubulin at the proximal compartment of the cilium under ciliated conditions [29] (Figure 3). In previous studies, polyglutamylation was shown to be influenced by a complete loss of TTC30/IFT70 [17]. Interestingly, ciliary length and the level of polyglutamylated tubulin in TTC30A or TTC30B KO cell lines were mildly but significantly reduced compared to control cells. The number of cells harboring cilia remained unchanged (Supplementary Figure S3). The average ciliary length in control cells was 4.097 µm ± 0.055 µm; in TTC30A KO cells—3.363 µm ± 0.048 µm; and in TTC30B KO cells—3.122 µm ± 0.041 µm (n_control_ = 308; n_TTC30AKO_ = 224; n_TTC30BKO_ = 245). On average, cilia in KO cells were approximately 1 µm shorter compared to control cells (TTC30A KO: 21.04%; TTC30B KO: 29.22% reduction). GT335 intensity in KO cells was even more strongly affected. The average intensity dropped from 1.053 relative intensity units (IU) ± 0.026 IU in control cells to 0.6590 IU ± 0.026 IU (62.58% in TTC30A KO) and 0.617 IU ± 0.034 IU (58.56% in TTC30B KO) (Figure 3). These effects were even more severe in TTC30A/B double-KO cells, where ciliary and tubulin marker cannot be distinguished from the background, showing that cilia formation was absent.

**Figure 4:**
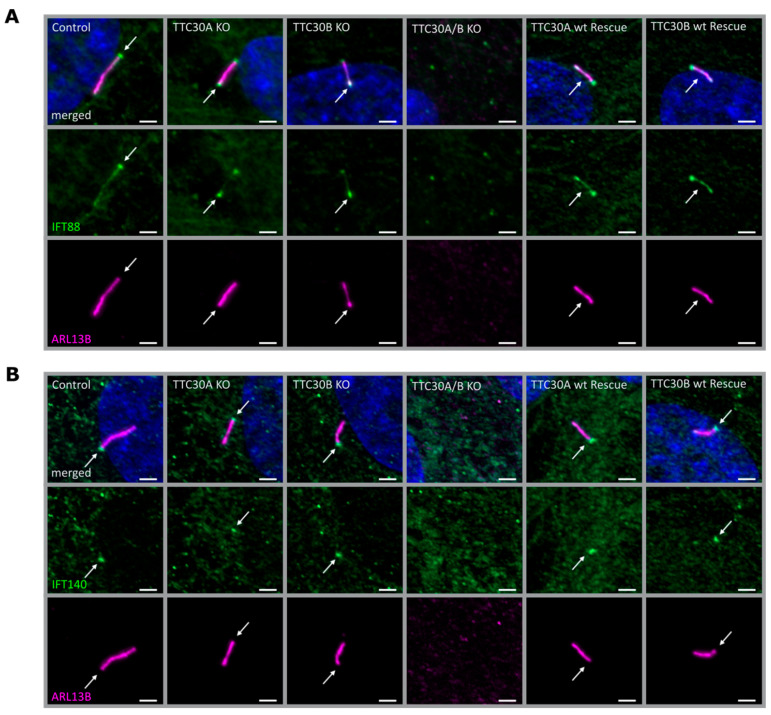
Fluorescent light microscopy images of hTERT-RPE1 control, TTC30A, TTC30B, TTC30A/B double-KO cells, TTC30A and TTC30B rescue (from left to right) are shown. The cells were stained for ARL13B (magenta), DNA is marked in dark blue and co-localization shown in white. The green channel depicts IFT88 (**A**) or IFT140 (**B**). The scale bar measures 2 µm.

**Figure 5:**
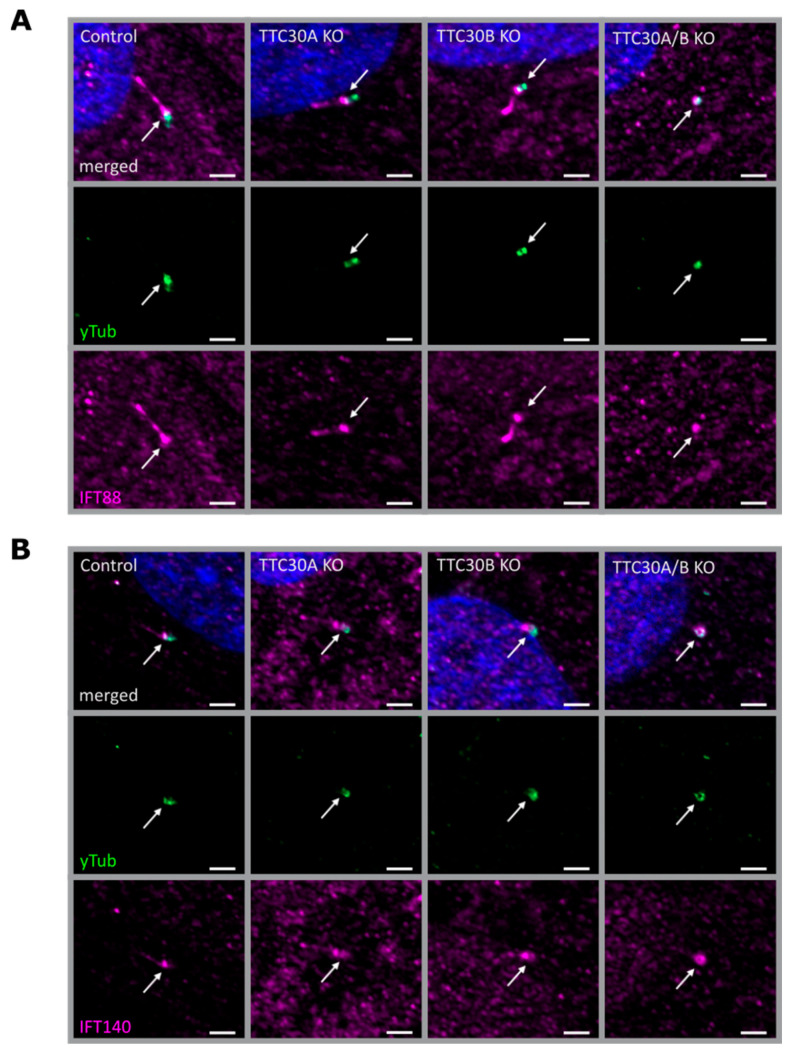
Fluorescent light microscopy pictures of hTERT-RPE1 control, TTC30A, TTC30B and TTC30A/B double-KO cells (from left to right) are shown. The cells were stained for γ-tubulin (green), DNA marked in dark blue and co-localization shown in white. The magenta channel depicts IFT88 (**A**) or IFT140 (**B**). The scale bar measures 2 µm.

Third, we evaluated whether a loss of TTC30 affected the ciliary localization of IFT complexes. Therefore, the IFT-B protein IFT88 and the IFT-A protein IFT140 were investigated in single- and double-KO cells, respectively (Figure 4). Interestingly, IFT88 and IFT140 localization was unaffected in both TTC30A and B single KO cell lines (Figure 4 and Figure 5). The total protein levels of IFT88/140 were investigated by measuring IFT88 and IFT140 intensities with normalization to the loading control (IFT88: n = 4; IFT140: n = 5). The overall intensities were slightly variable. Nevertheless, the resulting ratios were unchanged in single- and double-KO compared to control cells (Supplementary Figure S4).

To investigate the localization of IFT88 and IFT140 at the basal body, co-staining with γ-tubulin in hTERT-RPE1 control, TTC30A KO, TTC30B KO and TTC30A/B double-KO cells was performed. Both IFT proteins could be detected along the cilium and at the basal body in control and single-KO cells, whereas γ-tubulin was only located at the base. As Takei et al. showed, which we confirmed in the study presented here, IFT proteins still co-localize with γ-tubulin at the basal body in double-KO TTC30, implying that the mislocalization of IFT proteins is not the cause of the ciliogenesis defect [14] (Figure 5).

To prove that the phenotype described in the KO cells was specific for the loss of TTC30, rescue constructs were stably expressed in double-KO cells. After transient transfection, antibiotic treatment was applied to select stably transfected cells. The exogenously expressed TTC30 was detected in ciliated cells co-stained with ARL13B. Importantly, the loss of cilia formation in TTC30A/B double-KO cells was rescued by either TTC30A or TTC30B (Figure 2, Figure 3 and Figure 4). Ciliary length and intensities for polyglutamylated tubulin are comparable to those of TTC30A and TTC30B single-KO cells. In addition, the ciliary localization of IFT88 and IFT140 was rescued by expressing TTC30A or B (Figure 4). To elucidate the underlying mechanisms, which are common for both paralogues but also to shed light on distinct patterns, protein–protein interactions were analyzed for TTC30A and B separately.

### 3.2. TTC30A and TTC30B Interact with IFT-B Complex Components

**Figure 6:**
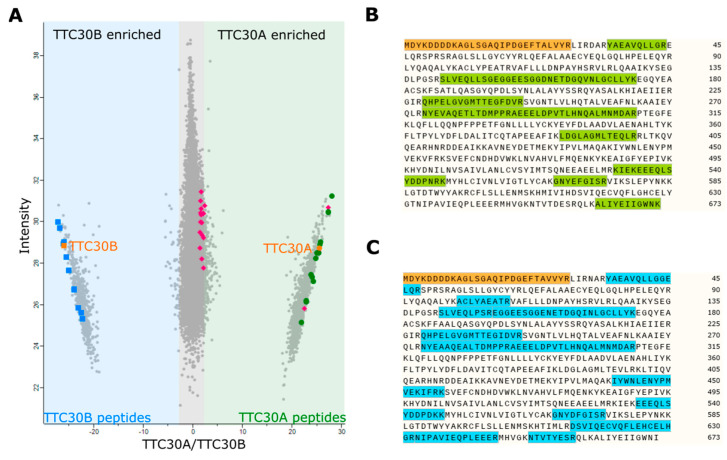
The scatter plot (**A**) depicts the distribution of all peptides detected. The x-axis depicts the log_2_ ratios of identified peptides and the y-axis the log_2_ of their respective intensities. Peptides occurring with higher abundance in TTC30A samples can be found on the right (**green**), whereas peptides with higher abundance in TTC30B samples on the left (**blue**). Peptides, which are equally abundant, can be found in the middle (**grey**). Specific and significantly enriched unique peptides are marked in green (TTC30A) and blue (TTC30B), and FLAG-tagged peptides in orange. Non-significant TTC30 peptides are marked in magenta. The amino acid sequences illustrate the specifically detected fragments of TTC30A ((**B**); **green**) and TTC30B ((**C**); **blue**) along with their FLAG-tagged peptides (**orange**) and the corresponding position in the protein sequence.

For the comparative analysis of the paralogues TTC30A and B, endogenously FLAG-tagged HEK293T cells were generated using CRISPR/Cas9. Preceding the analysis of TTC30A and B complexes, we aimed at verifying successful, paralogue-specific tagging. Comparative mass spectrometry revealed several peptides with an equal distribution in both samples (Figure 6). These protein fragments were assigned to both paralogues and are therefore not suitable for differentiation (22 peptides). However, specific peptides could be detected in either A (14 unique peptides) or B samples (11 unique peptides). The peptides unique for one or the other paralogue were significantly enriched in the corresponding samples (*t*-test, permutation-based FDR < 0.05, significance A, Benjamini–Hochberg FDR < 0.05; Supplementary Figure S2). These results demonstrate the successful and specific insertion of the FLAG-tag at the endogenous level for both paralogues, enabling a paralogue-specific interactome study (Figure 6).

**Figure 7:**
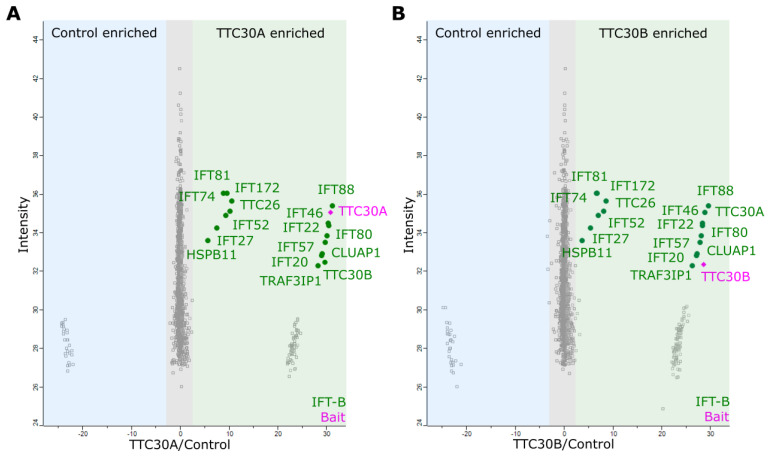
The scatter plots show the distribution of all proteins identified for TTC30A (**A**) as well as TTC30B (**B**). The x-axis depicts the log_2_ ratios and the y-axis shows the log_2_ of their respective intensities. The bait proteins TTC30A and B are shown in magenta and IFT complex B proteins in green. Proteins showing an increased binding to TTC30A/B are on the right compared to the control on the left.

Immunoprecipitation and a subsequent protein complex analysis were conducted by utilizing the same endogenously FLAG-tagged TTC30A and TTC30B HEK293T cells as used for the peptide specific validation approach above. A CRISPR/Cas9 empty vector treated and therefore untagged single clone was used as control. In total, six biological replicates for each condition were analyzed by mass spectrometry followed by identification and quantification using MaxQuant and statistical analysis using Perseus [30], https://maxquant.net/perseus/, version 1.6.2.3, accessed on 27 June.2022. The proteomics data were filtered to remove proteins only identified by site, reversed peptide sequences, or potential contaminants. Within the conditions, only proteins quantified in at least four out of six replicates were used for statistical analysis. An unpaired two-sample Student *t*-test (permutation-based FDR *p* < 0.05), to test result stability over the replicates, and an outlier test (significance A, Benjamini–Hochberg FDR *p* < 0.05) were applied. Proteins meeting the significance criteria were considered as being significantly enriched. For both TTC30A and B, all 16 IFT-B proteins were detected, proving the capability of each paralogue to bind to the IFT-B complex (Figure 7). Interestingly, not yet known interacting proteins were identified. Besides IFT-B, 30 proteins for TTC30A and 33 proteins for TTC30B were identified, with four being present in both datasets (Supplementary Table S1). However, 26 proteins only being detected with TTC30A and 29 proteins significantly enriched with TTC30B might hint towards non-overlapping and specific molecular functions for each paralogue individually.

### 3.3. IFT-B Complex Composition Was Disturbed by Loss of TTC30A and TTC30B

To better understand the strong phenotype observed in double compared to single-KO cells, the IFT-B complex composition was analyzed in TTC30B single- and TTC30A and TTC30B double-KO HEK293T cells. Successful CRISPR/Cas9-based KO generation was validated by Sanger sequencing (Supplementary Figure S5). For the purification of the IFT-B complex, two independent baits were used. Lebercilin (LCA5) is known to interact with IFT-B as well as IFT-A complex proteins without being an integral part of the complex [31,32]. IFT88 forms, together with IFT52, IFT46 and TTC30A/B, the essential linking complex to the IFT subcomplex B2 [12,14]. For affinity purification, Strep/FLAG-tag-containing constructs for LCA5, IFT88 and RAF1 as control were expressed in TTC30B single-, TTC30A/B double-KO and Cas9 empty HEK293T control cells [33]. Strep-based affinity purification was followed by quantitative mass spectrometry. The results of four biological replicates were filtered for a minimum of three valid values. Significantly enriched proteins comparing LCA5 or IFT88 to RAF1 control in each cell line (significance B < 0.05, paired *t*-test, permutation-based FDR corrected *p* < 0.05) were considered as specific interactors. These were comparatively analyzed in KO versus Cas9 control cells. The results are summarized in Table 1 and  Table S2.

As expected, TTC30B was not detected in TTC30A/B and TTC30B KO cells, but significantly enriched in Cas9 control cells with LCA5 or IFT88. TTC30A was highly enriched in TTC30B and Cas9 control cells. In TTC30A/B KO cells, a signal comparable to RAF1 with LCA5 and IFT88 was seen, which is most likely due to carry-over during sample preparation or mass spectrometry measurement.

With each bait, a significant decrease in IFT-B1 complex binding was observed when comparing protein–protein interactions detected in double-KO and Cas9 control cells, while IFT-B2 was mostly stable and showed no loss in binding. This might indicate instability in the link of IFT-B1-B2 due to the loss of TTC30. Interestingly, no change was detected in TTC30B single-KO cells, which is in accordance with the fact that a single KO is not sufficient to impede ciliogenesis.

## 4. Discussion

The initial question of whether TTC30A and TTC30B are two paralogues with redundant functions can only be answered if these proteins can be clearly distinguished from each other, which is challenging because their nucleotide sequences and amino acid sequences are almost identical (Supplementary Figure S1). Nevertheless, some regions show minor differences, which were sufficient for specific targeting. The sgRNA targets were located close to the N-terminus. Overall, they had only few predicted off-targets, which could be neglected due to their location in intergenic or intronic sequences with a minimum of three to four mismatches [34]. Finally, both proteins could be addressed due to the accuracy of the CRISPR/Cas9 method [35,36]. In addition, specific rescue restoring cilia assembly and polyglutamylation in the double-KO background to the single-KO ciliary length led to the conclusion that the effect seen in single- and double-KO cells was specific for TTC30. 

The results of the localization and interactome studies indicated that in TTC30A or TTC30B single-KO cells, the overall function of IFT complexes A and B is still intact. In contrast, the phenotype of double-KO TTC30A/B led to the assumption that IFT complex B is destabilized or not assembled, eventually leading to disrupted intraflagellar transport and, subsequently, no ciliary assembly. This hypothesis is supported by the complex instability seen in TTC30A/B double- but not TTC30B single-KO cells, with at least the subcomplex IFT-B1 being influenced by the loss of both paralogues simultaneously (Table 1). However, it is still up for speculation if the instability would result in transport defects. Interestingly, the strong phenotype observed in double-KO cells could be partially rescued by expressing TTC30A or TTC30B again. In conclusion, one TTC30 paralogue, due to redundancy, is enough to restore the integrity of the IFT complex, which is required for the induction of primary cilium assembly. This is in line with the results described by Takei and colleagues in 2018 [14]. 

Similar to the results obtained here, Takei and colleagues generated double-KO hTERT-RPE1 cells and were able to rescue cilia when expressing one paralogue only. However, in contrast to the published data, the use of the single-KO type revealed a mild cilia length reduction, which was not shown before and hints towards paralogue-specific functions. In addition, the paralogue-specific rescue restored cilia length to a level comparable to that of single-KO cells but not to the level of control cells, which was also true for polyglutamylated tubulin intensity (Figure 3). Interestingly, it was shown that IFT70/TTC30A/B is capable of binding IFT52-IFT88, which is required for ciliogenesis [8,12,14]. Takei and colleagues suggested that this binding induces conformational changes in the IFT-B complex which might be a prerequisite for ciliary transport. The mild change in IFT-B complex stability seen here in TTC30A/B double-KO cells supports this hypothesis. However, it is still unclear as to whether the reduction in cilia length is due to (1) ciliary tip instability, (2) transport deceleration, or (3) axoneme instability. Therefore, it was of interest to investigate paralogue-specific protein–protein interactions, which might help us to understand the mild effects seen in single-KO cells.

The aim of TTC30A/B interactome analysis at the endogenous level was to be as close as possible to physiological conditions. Endogenous tagging using the small FLAG tag, reducing false positive or false negative hits and low interference with protein folding and interaction ([37,38]), led to the identification of a specific interactome for each paralogue containing all IFT-B complex proteins. The most important step was to endogenously and specifically FLAG-tag TTC30A or B. Otherwise, potential interacting proteins could be matched to the wrong paralogue. By detecting unique peptides for TTC30A or B, which were found to be enriched in corresponding samples, we could clearly show that the specific tagging was successful and therefore could assign potential interactors correctly. As a result, all components of IFT complex B could be found to interact with TTC30A as well as TTC30B (Figure 7). From the identification of the whole complex, a central role in IFT-B for both proteins is suggested, as assumed before [8,12,14]. Redundancy might be necessary for correct complex composition and integrity, even if one paralogue is mutated. Interestingly, unique TTC30A or B peptides were found in the related sample, indicating that the paralogues might compete for IFT-B complex binding (Table 1 and Table S1). Furthermore, some proteins were found to interact with either TTC30A or B, which hints towards specific roles probably influencing cilia length control or stability. TTC30A specifically interacts with CWC27, HAGH, RPP25L, CD3EAP, CARS, PPP1R12A and DHX34. To our knowledge, no ciliary function has been described for most of these proteins so far. CWC27 was linked to ciliopathy-related symptoms such as retinal degeneration, as well as skeletal and neurological defects [39]. TTC30B interacts with CROCC/rootletin and proteins of the CT45 family. CT45 proteins are not yet described to be involved in cilia functioning. However, rootletin is a protein important for centrosome cohesion and cilia stability [40,41]. In photoreceptors, mutant rootletin leads to photoreceptor instability and retinal degeneration [41]. *The Caenorhabditis* *elegans* ortholog *Che-10* is needed to support IFT-related transport and transition zone integrity [42]. However, in the study presented here, no effect on rootlet structures was seen (Supplemental Figure S6), but transport speed or transition zone integrity were not analyzed. NUP88, which did not pass the significance test in TTC30A samples but was detected with both paralogues, and FBXO3 are two candidates which are described to be involved in cilia function and might help to understand the mild effect seen in single-KO cells as well [43,44].

These findings underline, in combination with MS data, on the one hand, the need of these two paralogues with similar and non-overlapping functions, and on the other hand the importance of an intact IFT complex B itself. A loss of function in one protein can be to some degree compensated for, due to redundancy, by the other protein, but not completely. Cells still form a cilium but one that is shorter, and that is potentially limited in its functions. A complete loss of both proteins, such as the loss of other IFT proteins, leads to disrupted intraflagellar transport and, subsequently, no cilia formation at all, which is most likely a lethal condition during development [45,46,47]. Furthermore, these results show that both processes of polyglutamylation and ciliary assembly were affected by isoforms A and B, and therefore are similar to the phenotype found in zebrafish (*flr*), *C. elegans* (DYF1) [17] and *C. reinhardtii* [15]. Interestingly, there is no paralogue of TTC30 analogues DYF1/*flr* described that can compensate for phenotypes caused by mutation or complete loss. However, in more complex organisms (frogs, mice), paralogue TTC30 proteins can be found [22,48]. Therefore, gene duplication resulting in TTC30A and TTC30B might be an evolutionary asset worth conserving. More recently, in organisms with TTC30 paralogues new specific functions could be observed. In *Xenopus tropicalis*, TTC30A was linked to ciliary chondrodysplasia with polycystic kidney disease [48]. Furthermore, ciliary signaling could be linked to a rare TTC30B missense variant [13], leading to synpolydactyly. Intriguingly, synpolydactyly and chondrodysplasia are both skeletal phenotypes. In human cell lines, polyglutamylation, which is influenced by TTC30 orthologues [17], is a major contributor for ciliary signaling and suggests a potential therapeutic strategy by targeting polyglutamylation machinery. The aim is to promote the ciliary targeting of signaling machineries and correct signaling defects in ciliopathies [29,49]. However, the underlying mechanism of reduced polyglutamylation in cilia or mildly reduced ciliary length in humans remains unclear. The observed hypo-polyglutamylation might also be a result of the reduced ciliary length maintaining normal ciliary segmentation [50]. The mild reduction in ciliary length might hint towards a transport defect or some influence on ciliary function independent of IFT. Transmembrane protein interactors, such as TMEM41B, were found, which show a ciliary effect and are linked to TTC30B [8]. In addition, in the study presented here, more than 30 proteins were found to be specifically enriched for either TTC30A or B. These proteins hint at further functions that need to be elucidated using tissue-specific TTC30A and B investigations evaluating its function in motile and primary cilia. 

## Figures and Tables

**Table 1:** IFT-B1 protein binding was impaired in TTC30A/B double-KO cells. Strep-based affinity purification of overexpressed LCA5, IFT88 or RAF1 as control was performed in TTC30A/B KO, TTC30B KO and Cas9 control cells, respectively. Here, the log_2_ median values of four biological replicates (six columns to the right) and the calculated ratio (two columns to the left) for all 16 IFT-B proteins and LCA5 are shown. All proteins shown were significantly enriched when compared to RAF1 except HSPB11 and TTC26, which did not pass the t-test, but are included for completeness (significance B < 0.05, students *t*-test *p* < 0.05). IFT-B2 proteins are depicted in blue. Values ranging between −1 and −30 are highlighted in red; +1 and +40 in green.

Bait		LCA5		RAF1			LCA5		
	Cell Line	TTC30A/B KO versus Control	TTC30B KO versus Control	TTC30 A/B KO	TTC30 B KO	Cas Control	TTC30 A/B KO	TTC30 B KO	Cas Control
Gene Name									
*TTC30B*		−28.42	−28.42	0.00	0.00	0.00	0.00	0.00	28.42
*TTC30A*		−10.88	0.42	0.00	0.00	22.36	19.87	31.17	30.75
*IFT52*		−1.13	0.32	0.00	0.00	20.46	29.69	31.14	30.82
*IFT46*		−1.04	0.45	21.56	21.85	23.18	29.37	30.87	30.42
*IFT81*		−1.44	0.14	20.64	23.03	22.58	30.79	32.37	32.24
*IFT74*		−1.32	0.14	24.04	24.01	24.30	30.86	32.32	32.18
*IFT88*		−1.27	0.57	19.93	0.00	23.55	30.16	32.00	31.43
*TTC26*		−1.24	0.21	23.99	23.94	24.05	29.66	31.12	30.91
*IFT27*		−1.03	0.17	25.32	25.19	25.27	29.51	30.71	30.54
*IFT22*		−1.02	0.26	0.00	23.17	22.89	29.69	30.97	30.71
*HSPB11*		−0.94	0.26	24.00	23.97	24.24	28.20	29.40	29.13
*TRAF3IP1*		−0.34	0.88	23.45	22.69	21.40	29.91	31.13	30.24
*CLUAP1*		−0.33	0.43	0.00	0.00	18.33	31.27	32.03	31.60
*IFT57*		−0.27	0.46	21.63	21.16	20.58	31.54	32.27	31.81
*IFT80*		−0.20	0.53	18.97	0.00	0.00	31.96	32.70	32.16
*IFT172*		−0.18	0.46	24.82	24.41	24.62	34.01	34.64	34.18
*IFT20*		−0.06	0.60	0.00	0.00	0.00	30.93	31.60	31.00
*LCA5*		0.47	0.42	24.86	24.98	24.46	37.55	37.50	37.08
**Bait**		**IFT88**		**RAF1**			**IFT88**		
	**Cell Line**	**TTC30A/B KO** **versus Control**	**TTC30B KO** **versus Control**	**TTC30** **A/B KO**	**TTC30** **B KO**	**Cas** **Control**	**TTC30** **A/B KO**	**TTC30** **B KO**	**Cas** **Control**
**Gene Name**									
*TTC30B*		−26.77	−26.77	0.00	0.00	0.00	0.00	0.00	26.77
*TTC30A*		−7.47	−0.67	0.00	0.00	22.36	21.64	28.44	29.11
*IFT52*		−1.22	−0.60	0.00	0.00	20.46	27.58	28.20	28.80
*IFT46*		−1.19	−0.59	21.56	21.85	23.18	27.44	28.04	28.63
*IFT81*		−1.34	−0.67	20.64	23.03	22.58	28.77	29.45	30.11
*IFT74*		−1.49	−0.80	24.04	24.01	24.30	28.69	29.38	30.18
*IFT88*		−0.40	−0.46	19.93	0.00	23.55	34.18	34.11	34.57
*TTC26*		−1.09	−0.59	23.99	23.94	24.05	27.86	28.36	28.94
*IFT27*		−0.98	−0.67	25.32	25.19	25.27	27.68	28.00	28.67
*IFT22*		−1.29	−0.81	0.00	23.17	22.89	27.55	28.03	28.85
*HSPB11*		−0.94	−0.61	24.00	23.97	24.24	26.43	26.76	27.37
*TRAF3IP1*		0.78	−0.03	23.45	22.69	21.40	25.57	24.75	24.79
*CLUAP1*		0.34	−0.21	0.00	0.00	18.33	26.22	25.67	25.88
*IFT57*		0.46	−0.24	21.63	21.16	20.58	26.74	26.03	26.28
*IFT80*		0.77	0.03	18.97	0.00	0.00	26.05	25.30	25.28
*IFT172*		0.41	−0.10	24.82	24.41	24.62	29.33	28.82	28.92
*IFT20*		0.56	−0.10	0.00	0.00	0.00	26.22	25.56	25.66
*LCA5*		0.75	−0.27	24.86	24.98	24.46	26.62	25.60	25.88

## Data Availability

The mass spectrometry proteomics data have been deposited to the ProteomeXchange Consortium via the PRIDE partner repository with the dataset identifier PXD034412 [53].

## Supplementary Materials

The following supporting information can be downloaded at: https://www.mdpi.com/article/10.3390/genes13071191/s1. Figure S1: Alignment of TTC30A and B nucleotide sequences [51,52]; Figure S2: MaxQuant settings; Figure S3: Ciliation of hTERT-RPE1 KO cells; Figure S4: Protein level of IFT70/88/140 and acetylated Tubulin; Figure S5: Sequencing results for HEK293T TTC30A/B single and double KO; Figure S6: Localization of ARL13B and Rootletin in control and KO cells; Table S1: TTC30 interactome; Table S2: IFT88 and LCA5 interactome in KO cells.
