# Supplementary material for: TTC30A and TTC30B Redundancy Protects IFT Complex B Integrity and Its Pivotal Role in Ciliogenesis
Source: Genes (Basel). 2022 Jul 1;13(7):1191. doi: 10.3390/genes13071191 (PMC9319246; doi:10.3390/genes13071191)
Supplement: Supplementary file 1 [file genes-13-01191-s001.zip › Supplementary figure S1.pdf]

Supplementary Figure S1

I. Alignment of TTC30A and B nucleotide sequences

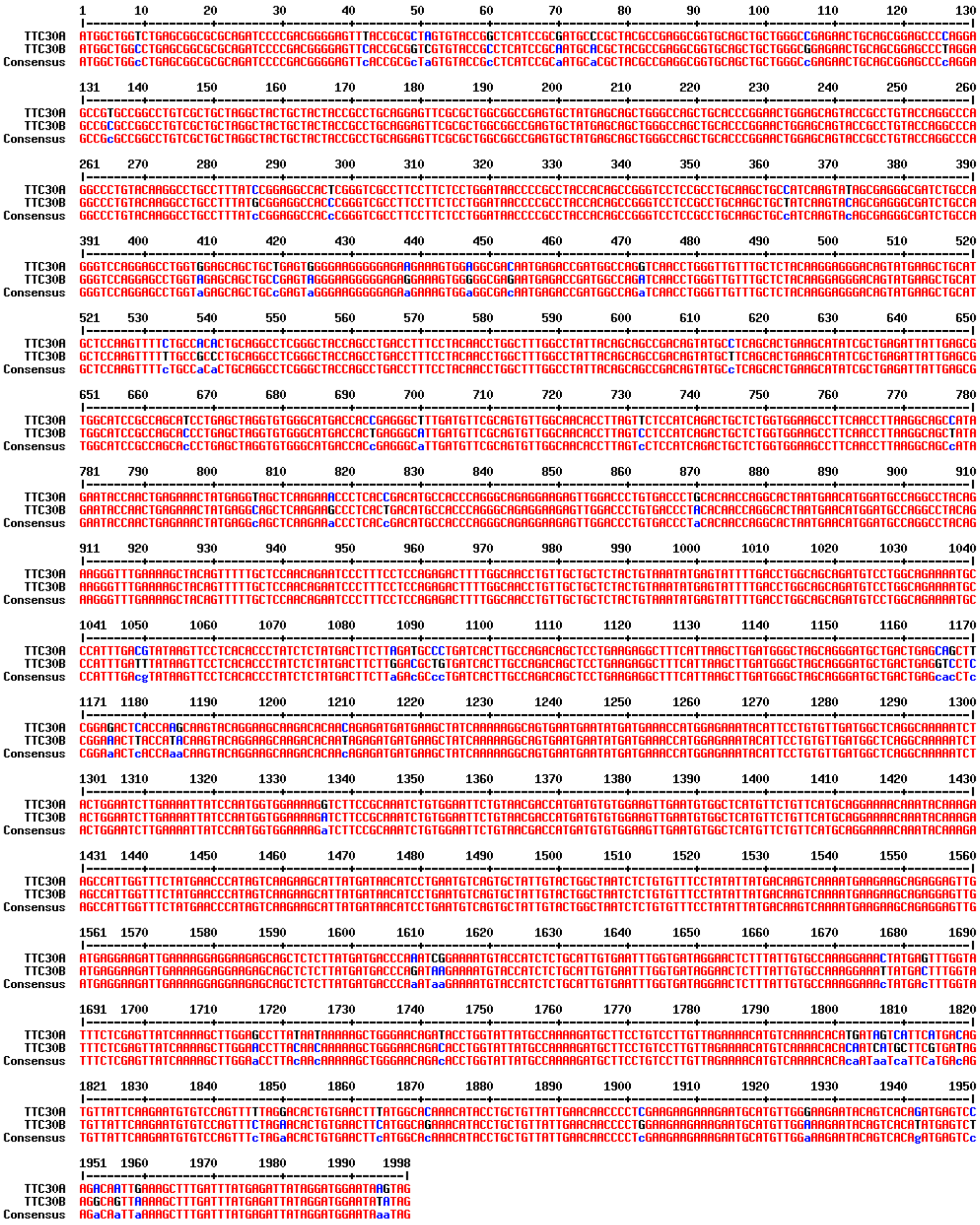

Alignment of TTC30A and TTC30B nucleotide sequences using multiple sequence alignment tool (Corpet, 1988). Different base pairs are depicted in blue, similarities in red.

II. TTC30A and B sequences showing sites of and premature stop

Translated sequence

```
wt   atg gct ggc ctg agc ggc gcg cag atc ccc gac ggg gag ttc acc gcg gtc gtg tac cgc
      M   A   G   L   S   G   A   Q   I   P   D   G   E   F   T   A   V   V   Y   R
     ctc atc cgc aat gca cgc.tac gcc gag gcg gtg cag ctg ctg ggc gga gaa ctg cag cgg
      L   I   R   N   A   R   Y   A   E   A   V   Q   L   L   G   G   E   L   Q   R
     agc cct agg agc cgc gcc ggc ctg tcg ctg cta ggc tac tgc tac tac cgc ctg cag gag
      S   P   R   S   R   A   G   L   S   L   L   G   Y   C   Y   Y   R   L   Q   E

KO   atg gct ggc ctg agc gca atg cac gct acg ccg agg cgg tgc agc tgc tgg gcg gag aac
      M   A   G   L   S   A   M   H   A   T   P   R   R   C   S   C   W   A   E   N
     tgc agc gga gcc cta gga gcc gcg ccg gcc tgt cgc tgc tag gct act gct act acc gcc
      C   S   G   A   L   G   A   A   P   A   C   R   C   -   A   T   A   T   T   A
     tgc agg agt tcg cgc tgg cgg
      C   R   S   S   R   W   R
```

Translated sequence

```
wt   atg gct ggt ctg agc ggc gcg cag atc ccc gac ggg gag ttt acc gcg cta gtg tac cgg
      M   A   G   L   S   G   A   Q   I   P   D   G   E   F   T   A   L   V   Y   R
     ctc atc cgc gat gcc cgc tac gcc gag gcg gtg cag ctg ctg ggc cga gaa ctg cag cgg
      L   I   R   D   A   R   Y   A   E   A   V   Q   L   L   G   R   E   L   Q   R
     agc ccc agg agc cgt gcc ggc ctg tcg ctg cta ggc tac tgc tac tac cgc ctg cag gag
      S   P   R   S   R   A   G   L   S   L   L   G   Y   C   Y   Y   R   L   Q   E
     ttc gcg ctg gcg gcc gag
      F   A   L   A   A   E

KO   atg gct ggt ctg agc ggc gcg cag atc ccc gac gta ccg gct cat ccg cga tgc ccg cta
      M   A   G   L   S   G   A   Q   I   P   D   V   P   A   H   P   R   C   P   L
     cgc cga ggc ggt gca gct gct ggg ccg aga act gca gcg gag ccc cag gag ccg tgc cgg
      R   R   G   G   A   A   A   G   P   R   T   A   A   E   P   Q   E   P   C   R
     cct gtc gct gct agg cta ctg cta cta ccg cct gca gga gtt cgc gct ggc ggc cga gtg
      P   V   A   A   R   L   L   L   L   P   P   A   G   V   R   A   G   G   R   V
     cta tga gca gct ggg cca
      L   -   A   A   G   P
```

The figure depicts parts of nucleotide as well as corresponding amino acid sequence of TTC30A (top) and TTC30B (bottom) wildtype (wt) and knockout (KO) cells. The sequences starting at N-terminus and the red boxes demonstrating first the location of the CRISPR/Cas9 mediated deletion and second the premature stop codon few amino acids later. Translation and depiction was done with Expasy translation tool (Duvaud et al., 2021).

III. Primer and sgRNA sequences

sgRNA	Nucleotide sequence
**TTC30A #1 Top**	TAGCGCGGTAAACTCCCCGT
**TTC30A #1 Bottom**	ACGGGGAGTTTACCGCGCTA
**TTC30A #2 Top**	AGTTTACCGCGCTAGTGTAC
**TTC30A #2 Bottom**	GTACACTAGCGCGGTAAACT
**TTC30B #1 Top**	CGGGGATCTGCGCGCCGCTC
**TTC30B #1 Bottom**	GAGCGGCGCGCAGATCCCCG
**TTC30B #2 Top**	CGTAGCGTGCATTGCGGATG
**TTC30B #2 Bottom**	CATCCGCAATGCACGCTACG
**HDR construct**	TTGCTCTGTTGCCATAGTAACCGCACCCATAACAGCCGTGGTGGTTATGGATTATA

	AAGATGATGATGATAAAGCTGGCCTTAGCGGTCGCAGATCCCCGACGGGGAGTT CACCGCGGT
**Primer**	
**TTC30A forward**	CGTCTTTCCCCTTCTGACTGCC
**TTC30A reverse**	GCAGATCGCCCTCGCTATACTTG
**TTC30B forward**	GTAGTCTTTCCCCTCCTGAATG
**TTC30B reverse**	ATTGCGGATGAGGCGGTACAC
**Flag tag forward**	ATGGATTATAAAGATGATGATGATAAA

The table shows the nucleotide sequences of the used Primers,sgRNAs and the HDR construct (produced by Integrated DNA Technolgies, USA)
