# Supplementary material for: TTC30A and TTC30B Redundancy Protects IFT Complex B Integrity and Its Pivotal Role in Ciliogenesis
Source: Genes (Basel). 2022 Jul 1;13(7):1191. doi: 10.3390/genes13071191 (PMC9319246; doi:10.3390/genes13071191)
Supplement: Supplementary file 1 [file genes-13-01191-s001.zip › Supplementary figure S2.pdf]

Supplementary Figure S2

I. MaxQuant settings for TTC30A/B protein identification

PARAMETER VALUE
VERSION 1.6.0.16
USER NAME MPCADMIN
MACHINE NAME ADMIN-PC
DATE OF WRITING 02/16/2019 20:05:05
FIXED MODIFICATIONS
CARBAMIDOMETHYL (C)
INCLUDE CONTAMINANTS TRUE
PSM FDR 0.01
PROTEIN FDR 0.01
SITE FDR 0.01
USE NORMALIZED RATIOS FOR OCCUPANCY TRUE
MIN. PEPTIDE LENGTH 7
MIN. SCORE FOR UNMODIFIED PEPTIDES 0
MIN. SCORE FOR MODIFIED PEPTIDES
40
MIN. DELTA SCORE FOR UNMODIFIED PEPTIDES 0
MIN. DELTA SCORE FOR MODIFIED PEPTIDES 6
MIN. UNIQUE PEPTIDES 0
MIN. RAZOR PEPTIDES 1
MIN. PEPTIDES 1
USE ONLY UNMODIFIED PEPTIDES AND
FALSE
PEPTIDES USED FOR PROTEIN QUANTIFICATION RAZOR
DISCARD UNMODIFIED COUNTERPART PEPTIDES TRUE
LABEL MIN. RATIO COUNT 2
USE DELTA SCORE FALSE
IBAQ FALSE
IBAQ LOG FIT FALSE
MATCH BETWEEN RUNS TRUE
MATCHING TIME WINDOW [MIN]
0.7
ALIGNMENT TIME WINDOW [MIN]
20
FIND DEPENDENT PEPTIDES FALSE
FASTA FILE C:\DATABASES\SP-HUMAN-2014-11.FASTA
DECOY MODE REVERT
INCLUDE CONTAMINANTS TRUE
ADVANCED RATIOS TRUE
FIXED ANDROMEDA INDEX FOLDER
TEMPORARY FOLDER

Max. peptide mass [Da] 4600
Min. peptide length for unspecific search 8
Max. peptide length for unspecific search 25
Razor protein FDR True
Disable MD5 False
Max mods in site table 3
Match unidentified features True
MS/MS tol. (FTMS) 20 ppm
Top MS/MS peaks per Da interval. (FTMS) 12
Da interval. (FTMS) 100
MS/MS deisotoping (FTMS) True
MS/MS deisotoping tolerance (FTMS)
7
MS/MS deisotoping tolerance unit (FTMS) ppm
MS/MS higher charges (FTMS) True
MS/MS water loss (FTMS) True
MS/MS ammonia loss (FTMS) True
MS/MS dependent losses (FTMS)
True
MS/MS recalibration (FTMS) False
MS/MS tol. (ITMS) 0.5 Da
Top MS/MS peaks per Da interval. (ITMS) 8
Da interval. (ITMS) 100
MS/MS deisotoping (ITMS) False
MS/MS deisotoping tolerance (ITMS)
0.15
MS/MS deisotoping tolerance unit (ITMS) Da
MS/MS higher charges (ITMS) True
MS/MS water loss (ITMS) True
MS/MS ammonia loss (ITMS) True
MS/MS dependent losses (ITMS)
True
MS/MS recalibration (ITMS) False
MS/MS tol. (TOF) 40 ppm
Top MS/MS peaks per Da interval. (TOF)
10
Da interval. (TOF) 100
MS/MS deisotoping (TOF) True
MS/MS deisotoping tolerance (TOF)
0.01
MS/MS deisotoping tolerance unit (TOF) Da
MS/MS higher charges (TOF) True

COMBINED FOLDER LOCATION	MS/MS water loss (TOF) True
SECOND PEPTIDES TRUE	MS/MS ammonia loss (TOF) True
STABILIZE LARGE LFQ RATIOS TRUE	MS/MS dependent losses (TOF)True
SEPARATE LFQ IN PARAMETER GROUPS FALSE	MS/MS recalibration (TOF) False
REQUIRE MS/MS FOR LFQ COMPARISONS FALSE	MS/MS tol. (Unknown) 0.5 Da
CALCULATE PEAK PROPERTIES FALSE	Top MS/MS peaks per Da interval. (Unknown) 8
MAIN SEARCH MAX. COMBINATIONS 200	Da interval. (Unknown) 100
ADVANCED SITE INTENSITIES TRUE	MS/MS deisotoping (Unknown)False
LFQ NORM FOR SITES AND PEPTIDES FALSE	MS/MS deisotoping tolerance (Unknown) 0.15
WRITE MSSCANS TABLE TRUE	MS/MS deisotoping tolerance unit (Unknown) Da
WRITE MSMSSCANS TABLE TRUE	MS/MS higher charges (Unknown) True
WRITE MS3SCANS TABLE FALSE	MS/MS water loss (Unknown) True
WRITE ALLPEPTIDES TABLE TRUE	MS/MS ammonia loss (Unknown) True
WRITE MZRANGE TABLE FALSE	MS/MS dependent losses (Unknown) True
WRITE PASEFMSMSSCANS TABLE FALSE	MS/MS recalibration (Unknown) False
WRITE ACCUMULATEDPASEFMSMSSCANS TABLE FALSE	Site tables Oxidation (M)Sites.txt

An overview of the MaxQuant settings used for quantitative analysis of acquired mass spectrometry data sets.

II. Specific TTC30A/B peptide sequences and ratios

peptide sequence	specifity	TTC30A_x/y_TTC30B
AGLSGAQIPDGEFTALVYR	TTC30A	25.64221
ALIYEIIGWNK	TTC30A	26.56001
EEEQLSYDDPNR	TTC30A	23.57260
EEEQLSYDDPNRK	TTC30A	26.29461
GNYEFGISR	TTC30A	27.89663
IEKEEEQLSYDDPNR	TTC30A	22.45543
IEKEEEQLSYDDPNRK	TTC30A	23.60411
KIEKEEEQLSYDDPNR	TTC30A	25.73300
LDGLAGMLTEQLR	TTC30A	24.61827
NYEVAQETLTDMPPR	TTC30A	44.63029
NYEVAQETLTDMPPRAEEELDPVTLHNQALMNMDAR	TTC30A	24.72231
QHPELGVGMTTEGFDVR	TTC30A	27.93952
SLVEQLLSGEGGEESGGDNETDGQVNLGCLLYK	TTC30A	26.22704
YAEAVQLLGR	TTC30A	28.68934
ACLYAEATR	TTC30B	-25.61216
AGLSGAQIPDGEFTAVVYR	TTC30B	-22.61680
DSVIQECVQFLEHCELHGR	TTC30B	-26.44459
EEEQLSYDDPDKK	TTC30B	-22.61090
EGGEESGGENETDGQINLGCLLYK	TTC30B	-26.32410
GNYDFGISR	TTC30B	-25.62171
NIPAVIEQPLEEER	TTC30B	-27.35527
NTVTYESR	TTC30B	-24.41638
QHPELGVGMTTEGIDVR	TTC30B	-23.29323
SLVEQLPSR	TTC30B	-27.06034
YAEAVQLLGGELQR	TTC30B	-27.13242

III. IFT-B interactors of TTC30A/B and ratios

TTC30A interactors	TTC30A_x/y_Control	TTC30B interactors	TTC30A_x/y_Control
**TTC26**	31,51712	**TTC26**	29,65243
**IFT88**	31,63776	**IFT88**	30,08411
**TTC30A (Bait)**	31,50176	**TTC30A**	29,40869
**IFT20**	29,13248	**IFT20**	27,07368
**TTC30B**	29,87241	**TTC30B (Bait)**	29,02791
**TRAF3IP1**	28,57022	**TRAF3IP1**	26,43966
**IFT81**	8,99273	**IFT81**	6,49532
**CLUAP1**	29,19903	**CLUAP1**	27,06651
**IFT74**	9,26959	**IFT74**	7,23361
**IFT27**	5,88090	**IFT27**	3,95420
**IFT22**	30,72643	**IFT22**	28,68761
**IFT46**	30,86946	**IFT46**	28,92667
**IFT57**	29,75482	**IFT57**	27,47053
**IFT80**	30,11855	**IFT80**	27,81675
**IFT172**	12,52627	**IFT172**	10,40726
**IFT52**	10,05262	**IFT52**	8,18796
**HSPB11**	5,39968	**HSPB11**	3,51236
