# Supplementary material for: TTC30A and TTC30B Redundancy Protects IFT Complex B Integrity and Its Pivotal Role in Ciliogenesis
Source: Genes (Basel). 2022 Jul 1;13(7):1191. doi: 10.3390/genes13071191 (PMC9319246; doi:10.3390/genes13071191)
Supplement: Supplementary file 1 [file genes-13-01191-s001.zip › Supplementary figure S3.pdf]

Supplementary Figure S3

I. Ciliation of hTERT-RPE1 knockout cells compared to control cells

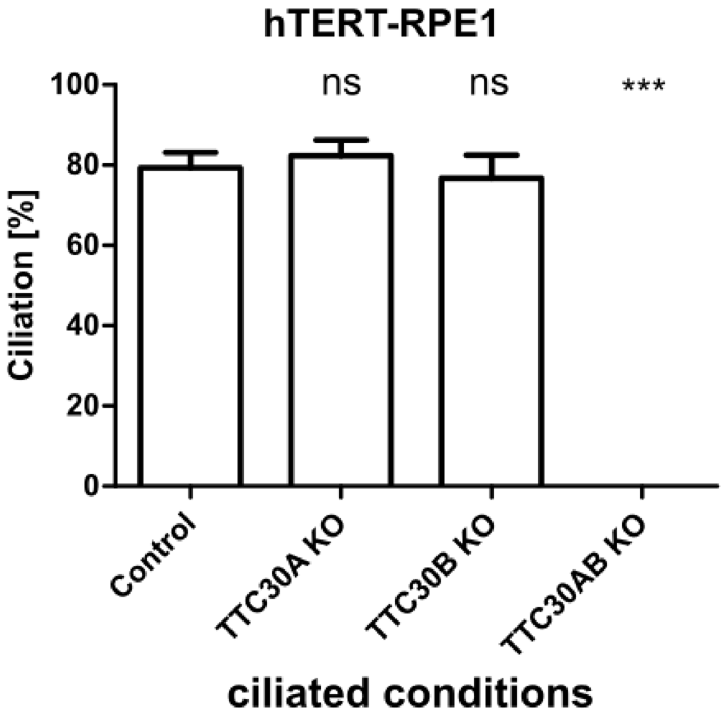

In hTERT-RPE1 TTC30A/B single and double knockout cells the number of cilia was counted and divided by the number nuclei obtaining the level of ciliation. P values below 0.001 are represented by ***. P values above 0.05 are determined not significant (ns). Error bars represent the s.e.m.
