# Supplementary material for: TTC30A and TTC30B Redundancy Protects IFT Complex B Integrity and Its Pivotal Role in Ciliogenesis
Source: Genes (Basel). 2022 Jul 1;13(7):1191. doi: 10.3390/genes13071191 (PMC9319246; doi:10.3390/genes13071191)
Supplement: Supplementary file 1 [file genes-13-01191-s001.zip › Supplementary figure S4.pdf]

Supplementary Figure S4

I. Protein level of IFT70/88/140 and acetylated Tubulin in hTERT-RPE1 knockout cells compared to control cells

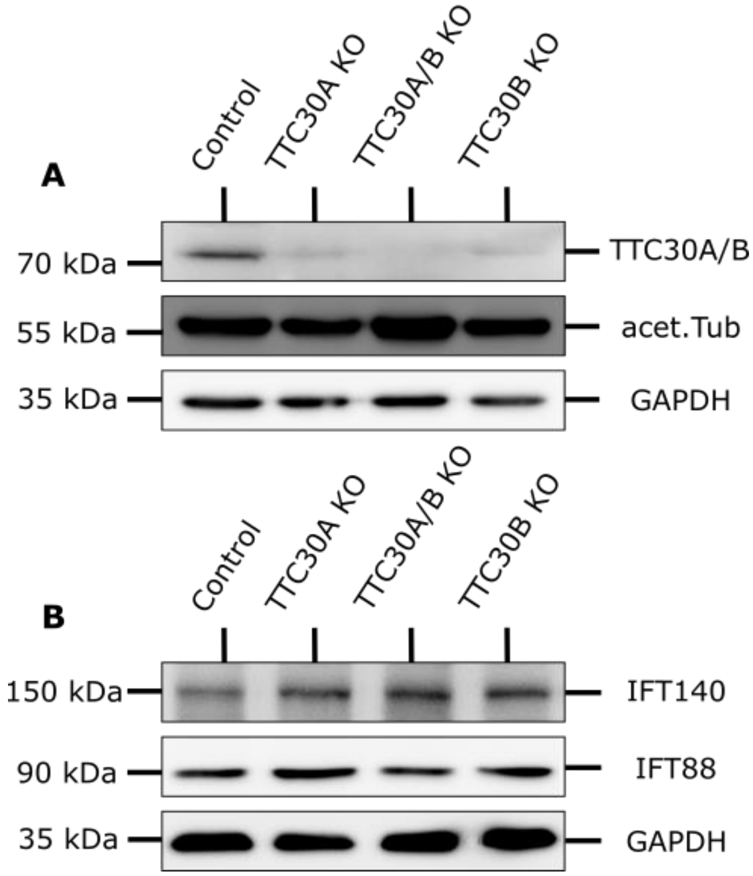

In hTERT-RPE1 TTC30A/B single and double knockout cells protein level of (A) TTC30A/B, acetylated tubulin and GAPDH as loading control were checked by Western Blot, as well as (B) IFT140, IFT88 and GAPDH.

II. Quantification of IFT88/140 protein level in hTERT-RPE1 knockout cells compared to control cells

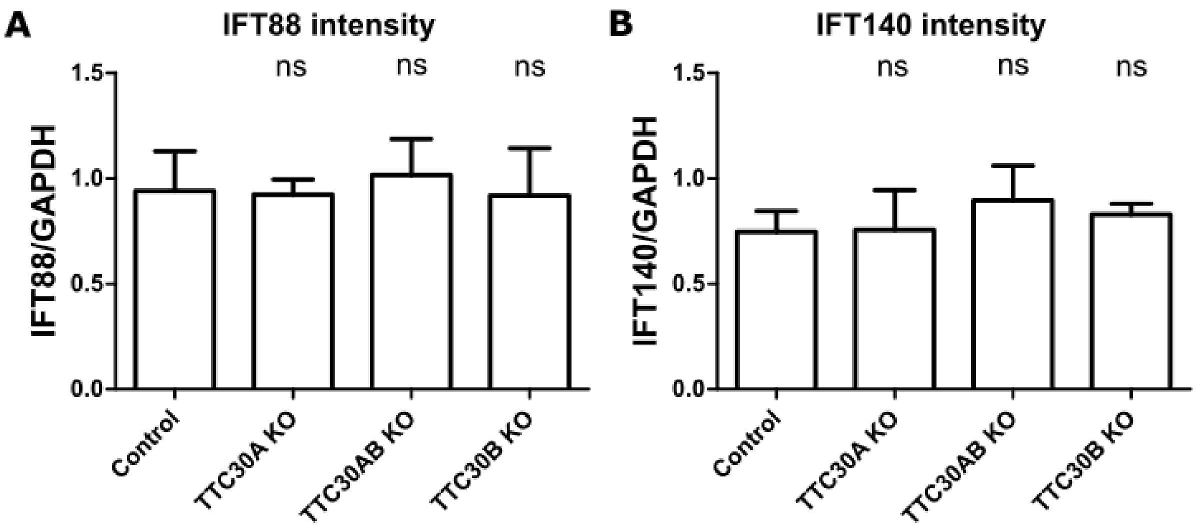

In hTERT-RPE1 TTC30A/B single and double knockout cells the protein level of IFT88 (A) and IFT140 (B) were quantified by measuring their intensity. The values were then normalized to the loading control (GAPDH). The resulting ratios of IFT88 (n = 4) as well as IFT140 (n = 5) for TTC30A, TTC30AB and TTC30B KO cells were comparable to those of control cells. For statistical analysis the mean was calculated. P values above 0.05 are deemed not significant (ns). Error bars represent the s.e.m.
