# Supplementary material for: TTC30A and TTC30B Redundancy Protects IFT Complex B Integrity and Its Pivotal Role in Ciliogenesis
Source: Genes (Basel). 2022 Jul 1;13(7):1191. doi: 10.3390/genes13071191 (PMC9319246; doi:10.3390/genes13071191)
Supplement: Supplementary file 1 [file genes-13-01191-s001.zip › Supplementary figure S5.pdf]

Supplementary Figure S5

I. Sequencing results for HEK293T TTC30A/B single and double knockouts

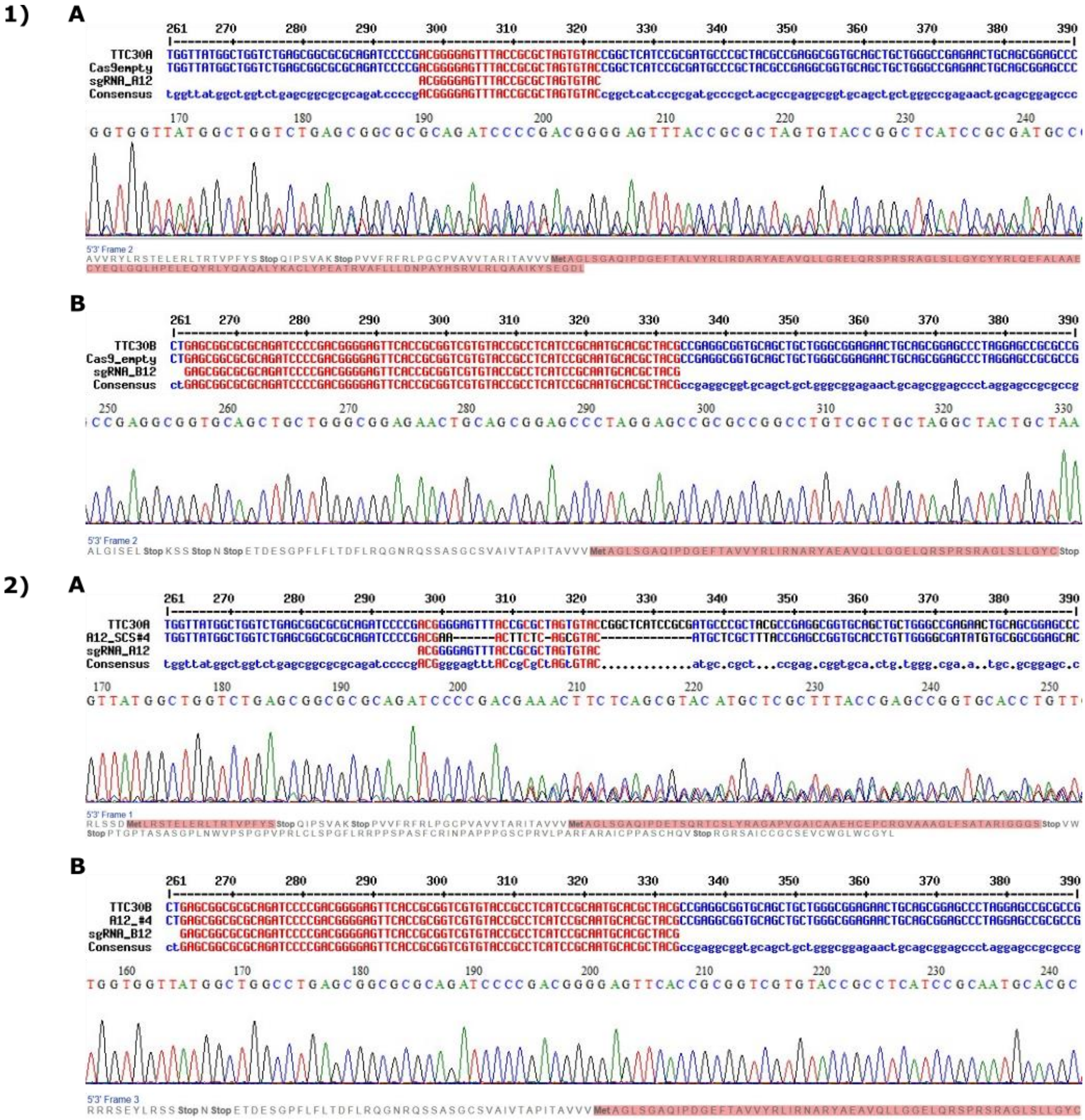

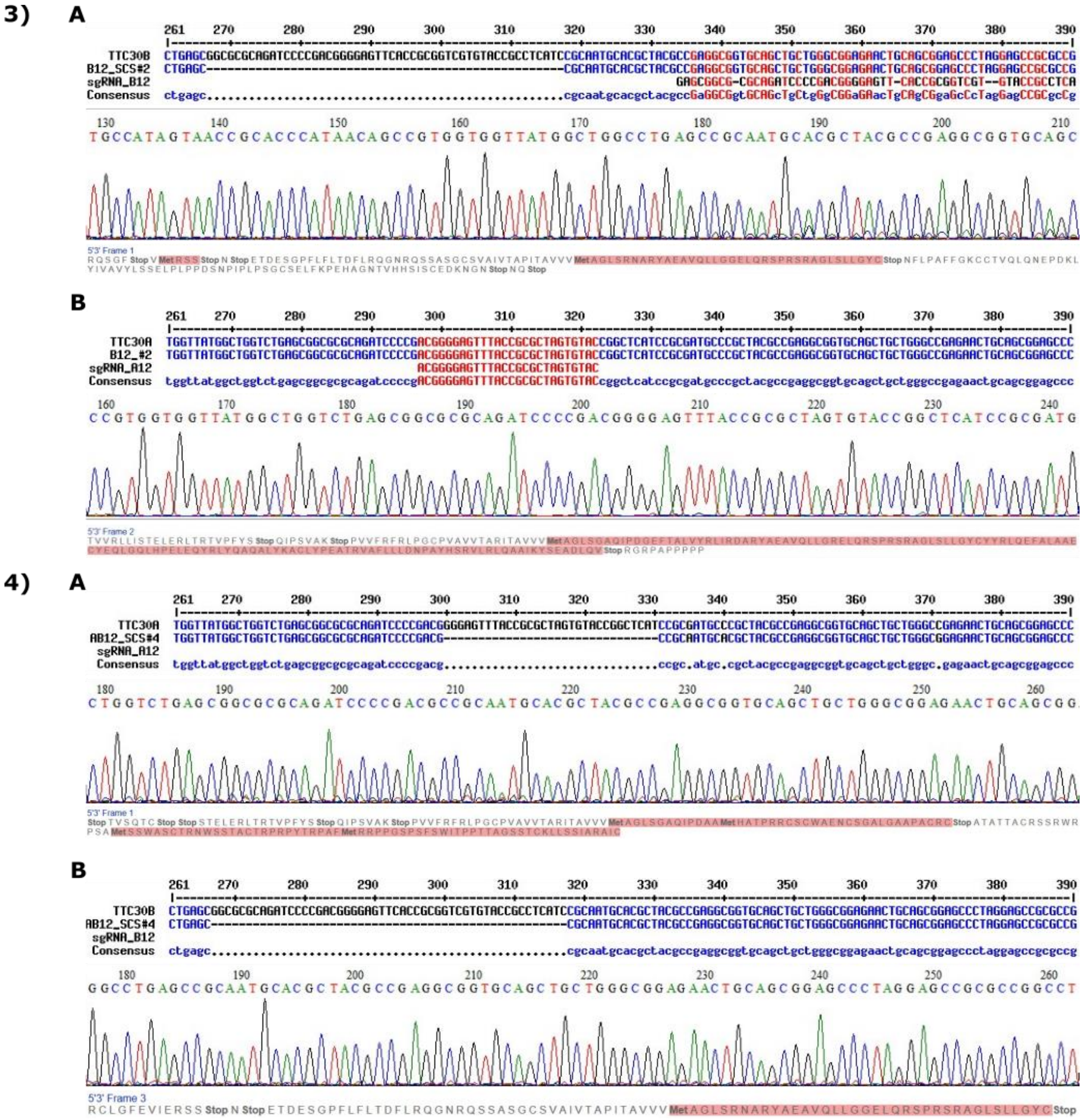

The figure depicts the results of (1) Cas9 empty treated HEK293T cells as well as (2) TTC30A knockout, (3) TTC30B knockout and (4) TTC30A/B double knockout cells sequenced for TTC30A (A) and TTC30B (B). Results include the alignment of TTC30 wildtype, TTC30 control or knockout sequences and used sgRNAs (top). Furthermore, the electropherogram (middle) of the respective knockout region and amino acid translation (bottom) depicting premature stop codons.
