# Supplementary material for: TTC30A and TTC30B Redundancy Protects IFT Complex B Integrity and Its Pivotal Role in Ciliogenesis
Source: Genes (Basel). 2022 Jul 1;13(7):1191. doi: 10.3390/genes13071191 (PMC9319246; doi:10.3390/genes13071191)
Supplement: Supplementary file 1 [file genes-13-01191-s001.zip › Supplementary figure S6.pdf]

Supplementary Figure S6

I. Localization studies of ARL13B and Rootletin in hTERT-RPE1 control and knockout cells

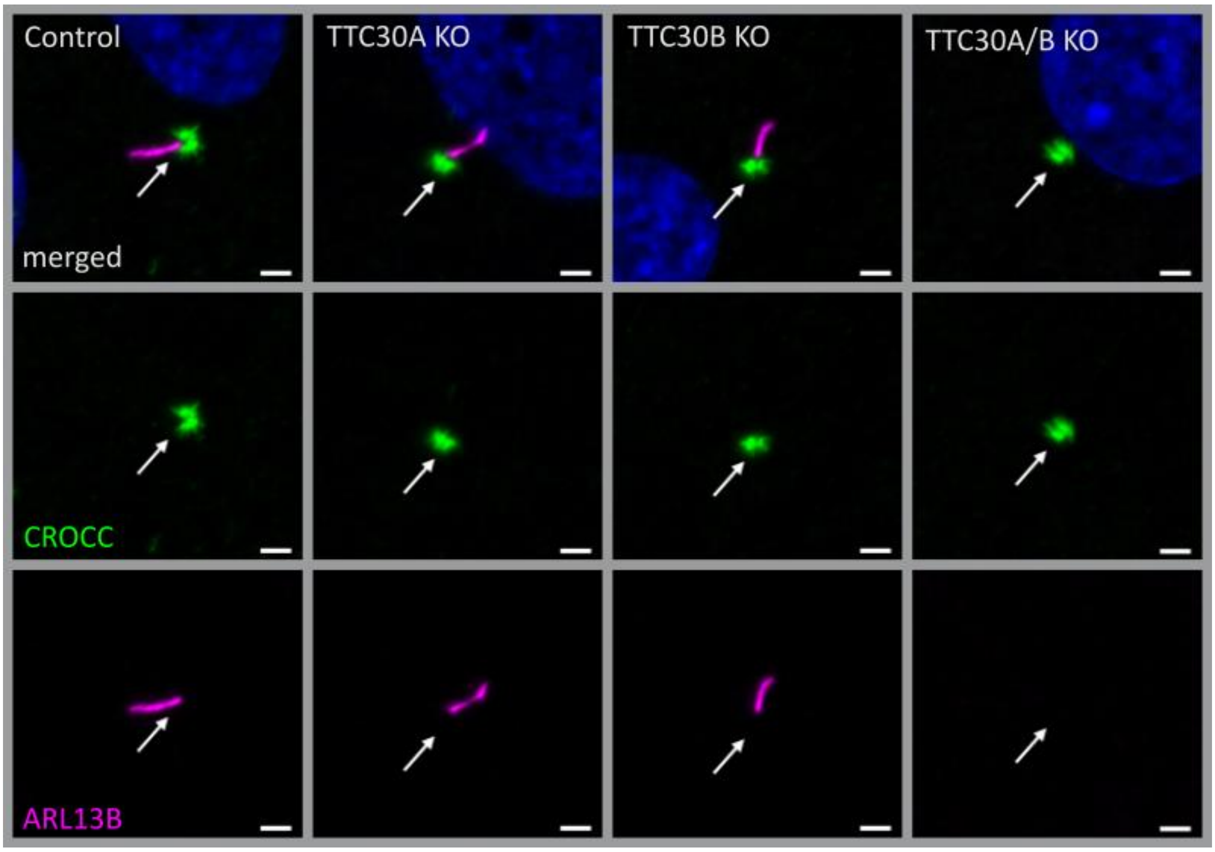

Fluorescent light microscopy pictures of hTERT-RPE1 control, TTC30A knockout, TTC30B knockout and TTC30A/B double knockout (from left to right) are shown. The cells were stained for ARL13B (magenta) and Rootletin (CROCC, green), DNA is marked in dark blue. The scale bar measures 2 µm.
